# Comparison of postoperative analgesic effects of erector spinae plane block and quadratus lumborum block in laparoscopic liver resection: study protocol for a randomized controlled trial

**DOI:** 10.1186/s13063-023-07341-w

**Published:** 2023-05-16

**Authors:** Yu Jeong Bang, Ji-Hye Kwon, RyungA Kang, Gaab Soo Kim, Ji Seon Jeong, Myungsuk Kim, Gyu-Seong Choi, Jong Man Kim, Justin Sangwook Ko

**Affiliations:** 1 Department of Anesthesiology and Pain Medicine, Samsung Medical Center, Sungkyunkwan University School of Medicine, 81 Irwon-Ro, Gangnam-Gu, Seoul, 06351 South Korea; 2 Department of Surgery, Samsung Medical Center, Sungkyunkwan University School of Medicine, 81 Irwon-Ro, Seoul, Gangnam-Gu 06351 South Korea

**Keywords:** Erector spinae plane block, Quadratus lumborum block, Pain, Postoperative, Hepatectomy, Laparoscopy

## Abstract

**Background:**

Compared with open surgery, laparoscopic liver resection is a minimally invasive surgical technique. However, a number of patients experience moderate-to-severe postoperative pain after laparoscopic liver resection. This study aims to compare the postoperative analgesic effects of erector spinae plane block (ESPB) and quadratus lumborum block (QLB) in patients undergoing laparoscopic liver resection.

**Methods:**

One hundred and fourteen patients undergoing laparoscopic liver resection will be randomly allocated to three groups (control, ESPB, or QLB) in a 1:1:1 ratio. In the control group, participants will receive systemic analgesia consisting of regular NSAIDs and fentanyl-based patient-controlled analgesia (PCA) according to the institutional postoperative analgesia protocol. In the two experimental groups (ESPB or QLB group), the participants will receive preoperative bilateral ESPB or bilateral QLB in addition to systemic analgesia according to the institutional protocol. ESPB will be performed at the 8th thoracic vertebra level with ultrasound guidance before surgery. QLB will be performed in the supine position on the posterior plane of the quadratus lumborum with ultrasound guidance before surgery. The primary outcome is cumulative opioid consumption 24 h after surgery. Secondary outcomes are cumulative opioid consumption, pain severity, opioid-related adverse events, and block-related adverse events at predetermined time points (24, 48, and 72 h after surgery). Differences in plasma ropivacaine concentrations in the ESPB and QLB groups would be investigated, and the quality of postoperative recovery among the groups will be compared.

**Discussion:**

This study will reveal the usefulness of ESPB and QLB in terms of postoperative analgesic efficacy and safety in patients undergoing laparoscopic liver resection. Additionally, the study results will provide information on the analgesic superiority of ESPB versus QLB in the same population.

**Trial registration:**

Prospectively registered with the Clinical Research Information Service on August 3, 2022; KCT0007599.

## Administrative information

Note: the numbers in curly brackets in this protocol refer to SPIRIT checklist item numbers. The order of the items has been modified to group similar items (see http://www.equator-network.org/reporting-guidelines/spirit-2013-statement-defining-standard-protocol-items-for-clinical-trials/).

**Table:** 

Title {1}	Comparison of postoperative analgesic effects of erector spinae plane block and quadratus lumborum block in laparoscopic liver resection: study protocol for a randomized controlled trial
Trial registration {2a and 2b}.	Prospectively registered with Clinical Research Information Service (https://cris.nih.go.kr/cris/search/detailSearch.do/23955) on August 3, 2022; KCT0007599.
Protocol version {3}	Protocol version 1.1 (July 22, 2022)
Funding {4}	This work was supported by the National Research Foundation of Korea (NRF) grant funded by the Korean government Ministry of Science and ICT (No. 2022R1F1A1074213)
Author details {5a}	Yu Jeong Bang^1^, Ji-Hye Kwon^1^, RyungA Kang^1^, Gaab Soo Kim^1^, Ji Seon Jeong^1^, Myungsuk Kim^1^, Gyu-Seong Choi^2^, Jong Man Kim^2^, Justin Sangwook Ko^1^ ^1^Department of Anesthesiology and Pain Medicine, Samsung medical center, Sungkyunkwan University School of medicine, Seoul, Korea ^2^Department of Surgery, Samsung Medical Center, Sungkyunkwan University School of Medicine, Seoul, Korea.
Name and contact information for the trial sponsor {5b}	The National Research Foundation of Korea (NRF). 201 Gajeong-ro, Yuseong-gu, Daejeon (34,113) Korea / TEL.82–42-869–6114
Role of sponsor {5c}	The sponsor and funding body were not involved in the study design; collection, analysis, and interpretation of data; the writing of the report; or the decision to submit the article for publication.

## Introduction

### Background and rationale {6a}

Laparoscopic liver resection is an increasingly common treatment option for the removal of benign masses or malignant tumors [1]. Laparoscopic liver resection has several advantages over open liver resection, including reduced pain and improved postoperative recovery [1–4]. However, a number of patients experience moderate-to-severe pain after laparoscopic liver resection [5]. For decades, opioids have been the mainstay of postoperative analgesia for this population. Although opioids provide excellent analgesia, opioid-related side effects, including nausea, vomiting, respiratory depression, and tolerance to analgesics are inevitably accompanied [6]. Reduced liver volume after liver resection can lead to delayed opioid metabolism and pronounced opioid-related side effects. Therefore, efforts should be made to reduce the opioid dose [7]. Recently, the Enhanced Recovery After Surgery (ERAS) Society recommended multimodal analgesia combined with intravenous (IV) opioids as the standard analgesia for laparoscopic liver resection [4]. Multimodal analgesia has been proposed to reduce opioid consumption while adequately addressing postoperative pain. It consists of several techniques and drugs with different mechanisms, including non-opioid analgesics (e.g., NSAIDs, acetaminophen), adjuvant anesthetics, and regional anesthesia [8]. Of these, regional anesthesia has emerged as a promising analgesic modality in this population [9], but relevant clinical data on analgesic efficacy and safety for each technique are lacking.

In our previous study comparing bilateral erector spinae muscle block (ESPB) with bilateral posterior quadratus lumborum block (QLB), we could not clearly demonstrate an analgesic difference between the groups [10]. This result may be attributed to the fact that we did not have a control group that received systemic analgesia alone or placebo. Another possible reason might be that the dose of local anesthetic (i.e., 40 mL of 0.375% ropivacaine) was too small to provide effective postoperative analgesia.

Therefore, in this study, we shall investigate the analgesic efficacy of ESPB or posterior QLB with 40 mL of 0.5% ropivacaine after laparoscopic liver resection compared with that in the control group. We will also measure chronological changes in plasma ropivacaine concentrations in both block groups to determine the safety of the local anesthetic dose in these patients. We hypothesize that ESPB or posterior QLB would reduce opioid consumption compared with conventional opioid-based analgesia in patients undergoing laparoscopic liver resection.

### Objectives {7}

We aim to evaluate the analgesic efficacy of the two interfascial blocks of ESPB and QLB in patients undergoing laparoscopic liver resection by comparing cumulative opioid consumption 24 h after surgery. We will investigate the differences in pain severity, cumulative opioid consumption, opioid-related side effects, and any block-related adverse events within 72 h after surgery among the groups. Postoperative recovery profiles will be also compared between the groups.

### Trial design {8}

This is a prospective, parallel-arm, double-blinded, superiority randomized controlled trial with 1:1:1 allocation.

## Methods: participants, interventions, and outcomes

### Study setting {9}

The present study will be conducted at Samsung Medical Center, a tertiary hospital located in Seoul, Republic of Korea.

### Eligibility criteria {10}

We shall enroll patients aged 19–69 years undergoing laparoscopic liver resection. The exclusion criteria are as follows: pregnancy, coagulopathy, cerebrovascular disease, systemic infection, inability to understand the study protocol, allergy to local anesthetics, psychopathy that may affect patient evaluation, and patient refusal.

### Who will take informed consent? {26a}

The research team members will invite eligible patients to participate in this study. The researchers (licensed medical doctors) will instruct them on the study protocol and obtain informed consent from all participants.

### Additional consent provisions for collection and use of participant data and biological specimens {26b}

Blood sampling will be performed only for 20 patients per group who receive ESPB or QLB at predetermined time points (30, 45, 60, and 240 min after administration of local anesthetics). The remaining blood samples will be discarded immediately after analysis of plasma ropivacaine concentration. We will obtain informed consent from all participants in advance.

## Interventions

### Explanation for the choice of comparators {6b}

The participants in the control group will be managed according to an institutional protocol.

The standardized anesthesia protocol is as follows: No premedication is given to any of the participants. Patient monitoring, including non-invasive blood pressure, EKG, pulse oximetry, and bispectral index (BIS), will be applied. After preoxygenation, general anesthesia will be induced with an IV injection of propofol (2 mg/kg) and rocuronium (0.8 mg/kg). After tracheal intubation, an arterial catheter is placed in the radial artery to continuously monitor blood pressure. Anesthesia is maintained with sevoflurane and IV infusion of remifentanil. The anesthetic concentration is adjusted to target BIS values of 40–50 and mean blood pressure and heart rate within 20% of the pre-induction values. IV hydromorphone (0.01 mg/kg) is injected 20 min before the end of surgery.

The standardized postoperative pain management protocol is as follows. After confirming full awakening from anesthesia, intravenous (IV) patient-controlled analgesia (PCA) is administered to the patient in the post-anesthesia care unit (PACU). The IV PCA consisting of fentanyl is programmed with a basal flow rate of 0.1 mL/h, bolus of 1 mL (15 μg), and a 15-min lockout interval. IV PCA is continued until postoperative day 3. In the ward, all patients are regularly administered IV ibuprofen 800 mg every 8 h. If patients present with breakthrough pain (NRS ≥ 4/10), IV hydromorphone (2 mg) is administered. Postoperative nausea and vomiting are treated with IV metoclopramide (10 mg).

### Intervention description {11a}

Except for the intervention of regional block and blood sampling for measuring plasma ropivacaine concentration, anesthesia, and postoperative pain management follow the same protocol as the control group. ESPB or QLB is performed after the induction of general anesthesia. These blocks will be performed by two experienced anesthesiologists (RAK and JSK). Intervention performers will not be involved in further data collection or outcome assessments. The procedure for each group is as follows:

- 1. ESPB group
  All participants in the ESPB group will receive bilateral ESPBs in the right lateral decubitus position. After confirming the 8th thoracic vertebra by ultrasound, a 21-gauge, 100-mm echogenic needle (SonoPlex®, PAJUNK®, Geisingen, Germany) will be inserted into the transverse process of the 8th thoracic vertebra in the cranial-to-caudal direction under ultrasound guidance. Right ESPB will be performed first, followed by left ESPB. The spread of local anesthetic is confirmed by lifting the erector spinae muscle plane from the transverse process. A total of 40 mL of 0.5% ropivacaine (20 mL on each side) will be administered to each participant.
- 2. QLB group
  All patients in the QLB group will receive bilateral QLBs in the supine position. After skin sterilization with 2% chlorhexidine, the same echogenic needle (SonoPlex®, PAJUNK®, Geisingen, Germany) will be inserted into the plane from the anterior-to-posterior direction and finally placed on the posterior surface of the quadratus lumborum under ultrasound guidance. The spread of local anesthetic is confirmed by pooling along the posterior aspect of the quadratus lumborum muscle. A total of 40 mL of 0.5% ropivacaine (20 mL on each side) will be administered to each participant.

In both the ESPB and QLB groups, blood samples will be collected from 20 patients in each group to determine plasma ropivacaine concentration. Through a pre-placed radial artery catheter, 5 mL of blood samples will be collected (i.e., a total of 20 mL) at predetermined time points (30 min, 45 min, 60 min, and 240 min after the end of both administrations of local anesthetics).

### Criteria for discontinuing or modifying allocated interventions {11b}

We will suspend the trial in cases of inappropriate spread of local anesthetic during ESPB or QLB, unexpected serious adverse events, and voluntary withdrawal of informed consent. These participants are considered as dropouts.

### Strategies to improve adherence to interventions {11c}

Not applicable. ESPB or QLB is performed with a single injection on the day of the surgery. Our institution’s standard protocol for anesthesia and postoperative pain management is strictly followed, regardless of the study. There will be no difficulty with non-adherence.

### Relevant concomitant care permitted or prohibited during the trial {11d}

Not applicable: No relevant concomitant care or intervention will be permitted or prohibited during the trial.

### Provisions for post-trial care {30}

According to the provisions of the compensation agreement, the investigator compensates the subject for physical damage caused by the intervention in a clinical trial through compensation insurance. Insurance also covers matters not included in the compensation agreement in accordance with the relevant laws of the Republic of Korea and the institutional regulations of Samsung Medical Center.

### Outcomes {12}

The primary outcome is cumulative opioid consumption 24 h after surgery. Secondary outcomes include: cumulative opioid consumption in the PACU (admission, highest, and discharge), at 48 h, and 72 h after surgery; pain severity at rest and when coughing in the PACU (admission, highest, and discharge), at 24 h, 48 h, and 72 h after surgery; postoperative nausea or vomiting in the PACU, at 24 h, 48 h, and 72 h after surgery; quality of sleep on the first night; patient satisfaction with pain relief at 24 h after surgery; postoperative recovery quality at 24 h after surgery using the Korean version of the Quality of Recovery-15 scale (QoR-15 K) [11]; the incidence of procedure-related complications (hematoma, infection, or needle trauma); time to first flatus; and plasma ropivacaine concentrations at 30 min, 45 min, 60 min, and 240 min after the end of both administrations of local anesthetics.

### Participant timeline {13}

**Fig. 1:**
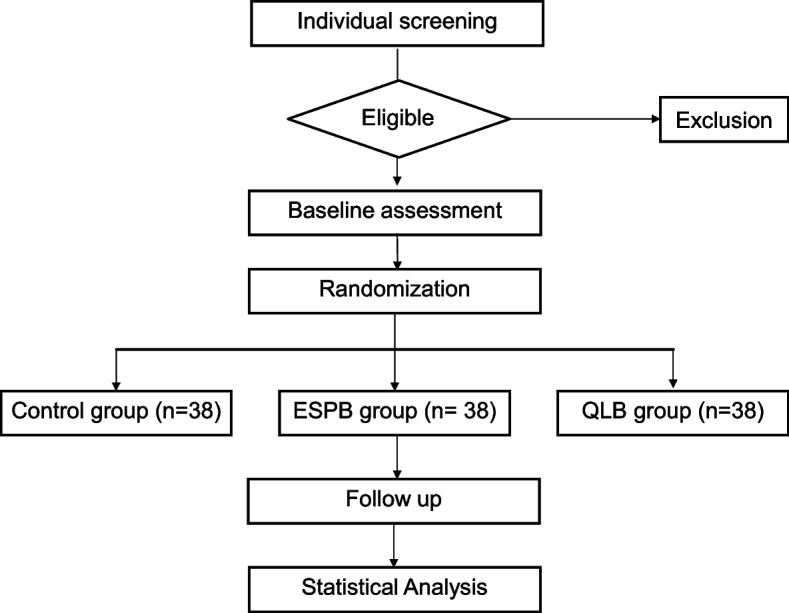
Flowchart of trial. ESPB, erector spinae plane block; QLB, quadratus lumborum block

**Fig. 2:**
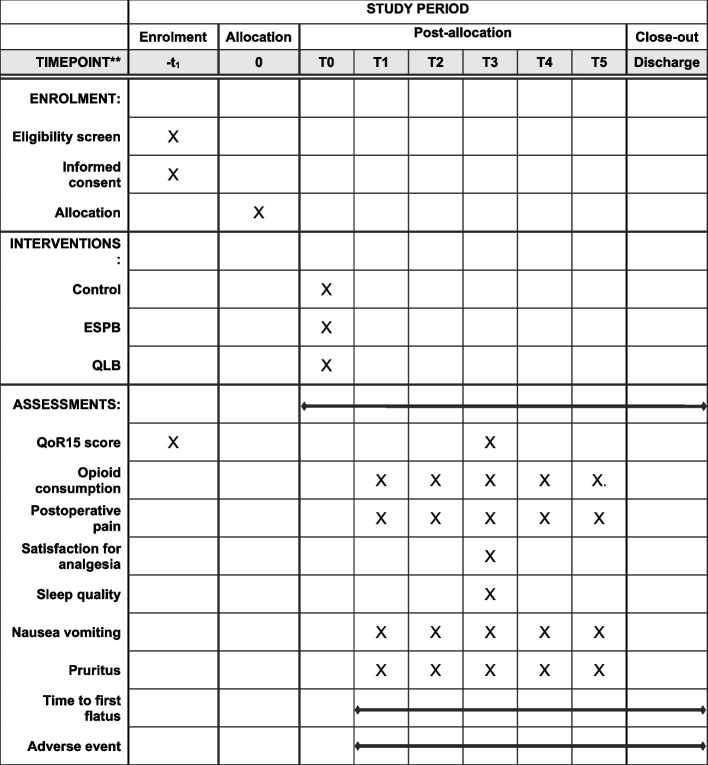
Schedule of enrolment, interventions, and assessments. − t_1_, the eve of surgery; T0, after induction of general anesthesia; T1, PACU admission; T2, PACU discharge; T3, 24 h after surgery; T4, 48 h after surgery; T5, 72 h after surgery

The schedule of enrollment, interventions, and assessments for the participants is shown in Figs. 1 and 2.

### Sample size {14}

The sample size was calculated based on a previous study and the retrospective clinical data from our institution [5]. The mean cumulative opioid consumption during 24 h after laparoscopic liver resection was 45.5 mg with a standard deviation (SD) of 21.8 mg in IV morphine equivalent dose (MED) [12]. We hypothesized that bilateral ESPB and QLB will reduce cumulative opioid consumption by 30% compared with the control group. With an alpha of 0.05 and a beta of 0.8, 34 participants are required in each group using Dunnett’s test. Assuming a dropout rate of 10%, we plan to enroll 114 participants in the study. The sample size was calculated using the PASS 2021 (NCSS, LLC. Kaysville, UT, USA).

### Recruitment {15}

Patients will be informed in detail of the course of the study, all study procedures, potential risks, and the benefits of each intervention one day before surgery. The chief investigator is responsible for participant recruitment. After screening adult patients aged 19–69 years who undergoing liver resection during the clinical trial period at Samsung Medical Center, enrollment will be performed using a consecutive sampling method on the eve of the surgery.

## Assignment of interventions: allocation

### Sequence generation {16a}

Eligible participants will be randomly assigned to the control, ESPB, or QLB group in a 1:1:1 ratio. Randomly assigned sequences are generated using a web-based service (www.sealedenvelope.com) by an independent statistician with a fixed block size of six.

### Concealment mechanism {16b}

The allocation information will be sealed in an opaque envelope and piled up in a cabinet in the research office. Only the independent statistician has access to the randomization list and allocation concealment. Randomization information will be managed confidentially such that the list is not disclosed to the evaluator.

### Implementation {16c}

The independent anesthetic nurse will open the sealed envelope of allocation information to confirm the assignment information on the operation day and prepare local anesthetics accordingly.

## Assignment of interventions: blinding

### Who will be blinded {17a}

All participants will be blinded because ESPB or QLB will be performed after the induction of general anesthesia. A blinded independent outcome assessor will collect the postoperative outcome data. The attending medical staff in the PACU and caregivers in the surgical ward will be blinded to the group allocation. The data analyst will be blinded to group allocation.

### Procedure for unblinding if needed {17b}

In case of medical necessity or emergency related to the procedure, the chief investigator could decide to unblind and inform the patient and attending medical staff.

## Data collection and management

### Plans for assessment and collection of outcomes {18a}

All interventions and outcome assessments will be performed by a medical doctor. The blinded outcome assessor will visit the patient five times after surgery (time at PACU admission and discharge, at 24 h, 48 h, and 72 h after surgery) and examine the patient. Opioid consumption will be collected cumulatively at each time point and presented as IV MED. To minimize missing values, the infusion information of PCA is automatically collected in the device log and exported to an Excel file. Pain severity will be assessed at designated times using a numerical rating scale (NRS, 0–10; 0 = no pain, 10 = worst imaginable pain). Opioid-related side effects including nausea, vomiting, and pruritus will be examined during 72 h after surgery using a 4-point scale (0–3; 0 = none, 1 = mild, 2 = moderate, 3 = severe). Twenty-four hours after surgery, all participants will be requested to rate their sleep quality and satisfaction with postoperative pain relief using a Likert scale (1–5; 1 = very dissatisfied, 2 = somewhat dissatisfied, 3 = neutral, 4 = somewhat satisfied, 5 = very satisfied). The QoR-15 K questionnaire will be measured preoperatively and 24 h postoperatively to assess the quality of postoperative recovery.

### Plans to promote participant retention and complete follow-up {18b}

Maximum effort will be made to engage participants in all assessments whenever possible and achieve high adherence in this study. The appointments for follow-up assessments will be provided to the participants in advance. The research team will conduct an assessment of the participants with careful consideration of their individual status.

### Data management {19}

Data will be recorded on a paper-based Case Report Form (CRF) and converted to an electronic CRF. The paper CRF will be locked into the storage of the research office. At least two members of the research team will prepare the electronic CRF, and the CRF will be checked for consistency every 20 enrollments.

### Confidentiality {27}

Personal data will be anonymized and identified using a predetermined numeric code in a randomization list. A link to personal data, including name and contact number, will be recorded in the subject identification log. The subject identification log will be stored in a separate locked storage in the research office.

### Plans for collection, laboratory evaluation, and storage of biological specimens for genetic or molecular analysis in this trial/future use {33}

Not applicable. We will perform blood sampling to measure plasma ropivacaine concentration. However, the blood samples will be anonymized prior to analysis, and no genetic or molecular analyses are planned in this study. The samples will be discarded immediately after analysis.

## Statistical methods

### Statistical methods for primary and secondary outcomes {20a}

After assessing normality, continuous variables will be presented as mean ± standard deviation or median with an interquartile range. Categorical variables will be presented as numbers (%).

The primary outcome, cumulative opioid consumption 24 h after surgery, will be compared using Dunnett's test. For continuous variables of the secondary outcomes, descriptive statistics for each group will be presented, and the mean difference among the groups will be confirmed using one-way analysis of variance (ANOVA). If the assumption of normality is not satisfied, the median difference among the groups will be analyzed using the Kruskal–Wallis test. For plasma ropivacaine concentration, the peak plasma ropivacaine concentration (Cmax) and time to Cmax (Tmax) will be compared using an independent two-sample *t*-test or Wilcoxon rank-sum test, depending on normality testing. For categorical variables, the difference in the ratio among the three groups will be confirmed using the chi-square test or Fisher’s exact test. Statistical significance shall be set at a two-sided *P* < 0.05. The analysis will be performed using SPSS, Version 27.0 (IBM Corp, Armonk, NY, USA).

### Interim analyses {21b}

Not applicable. An interim analysis is not planned for this trial.

### Methods for additional analyses (e.g., subgroup analyses) {20b}

Subgroup analyses will be performed for pairwise comparison. We will compare the primary outcome, cumulative opioid consumption at 24 h after surgery, between the two groups in each pair using an independent two-sample *t*-test or Wilcoxon rank-sum test as appropriate. Multiple comparisons for subgroup analysis will be adjusted using Bonferroni correction. One-way analysis of variance (ANOVA) with Tukey’s test will be used to perform pairwise comparisons for other outcomes. If the data do not satisfy normality, the Kruskal–Wallis test and Dunn’s method will be used to perform multiple comparisons.

### Methods in analysis to handle protocol non-adherence and any statistical methods to handle missing data {20c}

There will be no non-adherence issues in this study because the institutional standard protocol for postoperative pain management will be provided to all participants. The possibility of missing values in the primary outcome will be very low because data on opioid consumption will be collected using electronic medical records and PCA logs. Missing data will not be replaced.

### Plans to give access to the full protocol, participant-level data, and statistical code {31c}

Sharing participant-level dataset data can be considered by the corresponding author upon reasonable request.

## Oversight and monitoring

### Composition of the coordinating center and trial steering committee {5d}

The coordinating center is the Department of Anesthesiology and Pain Medicine of the Samsung Medical Center, Seoul, Korea. The trial steering committee consists of the chief investigator (RAK) and sub-investigators (YJB and JSK), who are responsible for recruiting patients, conducting the study, and data entry.

### Composition of the data monitoring committee, its role and reporting structure {21a}

The data quality of the trial will be evaluated by an independent data monitoring committee composed of the Samsung Medical Center Institutional Review Board, which is independent of the sponsor and competing interests. Data quality reporting will be conducted by the chief investigator.

### Adverse event reporting and harms {22}

The chief investigator will report any serious adverse events to each institutional review board within seven days of the notice. Mild adverse events and harm will be reported within 15 days of notice. When reporting adverse events or harm, we shall equally report causalities, time of occurrence, severity, seriousness, provided management, and the relationship with the present clinical trial.

### Frequency and plans for auditing trial conduct {23}

The research team will meet for self-auditing trial conduct every month. The trial will be conducted under the supervision of the Samsung Medical Center Institutional Review Board and may be audited at any time by the National Research Foundation of Korea.

### Plans for communicating important protocol amendments to relevant parties (e.g., trial participants, ethical committees) {25}

Any amendments to the protocol will be posted in the trial registry after approval by the Institutional Review Board.

### Dissemination plans {31a}

The results will be disseminated via publication in a peer-reviewed journal and may be presented at medical conferences.

## Discussion

For decades, opioid-based analgesia has been widely used to provide post-operative abdominal analgesia. Despite the excellent postoperative pain control of opioids, concerns have been raised regarding an increase in opioid-related side effects due to impaired metabolism of opioids after liver resection [7, 13]. In this regard, the ERAS protocol for liver resection [4] recommends multimodal analgesia combined with IV opioids as the standard analgesic for perioperative care during liver surgery and the limited use of systemic opioids as rescue analgesics [4, 14]. However, the use of non-opioid analgesics and adjuvant anesthetics is limited by the fixed maximum daily dose and therapeutic ceiling [15]. The regional anesthesia is relatively free from these concerns, compared with systemic analgesics. Accordingly, the latest Procedure-Specific Postoperative pain management (PROSPECT) guidelines for liver resection recommends that alternative analgesic techniques such as local anesthetic infiltration of surgical incisions or interfascial plane block would be helpful after liver resection [14]. However, there is a lack of evidence on what type of interfascial plane block should be performed in laparoscopic liver resection.

In this study, we chose the ESPB and posterior QLB as components of multimodal analgesia for the following reasons. First, both ESPB and QLB have been reported to provide postoperative abdominal analgesia in liver surgeries [10, 16–18]. The mechanism of action of ESPB is that injected local anesthetics spread to the paravertebral space and block the dorsal–ventral rami of the spinal nerve and sympathetic ganglia, providing somatic and visceral analgesia [19]. The mechanism of action of the QLB is that the injected local anesthetic spreads to the thoracoabdominal nerves [20]. Of the three approaches to QLB (anterior, lateral, and posterior), we chose posterior QLB, which provides a widespread sensory block of both somatic and visceral fibers from T7 to L1 or L2 levels [20]. Second, both the ESPB and QLB have been reported to be easy to perform and safe. EPSB is considered a superficial block with a low risk of bleeding or visceral injury because there are no critical structures in close proximity [21]. The QLB is considered a relatively deep fascial block [22], requiring caution, as the branches of the lumbar artery and intra-abdominal viscera are located adjacent to the needle trajectory [20]. However, the posterior QLB is performed in the most superficial plane of the three approaches. The needle tip is separated from the peritoneum by the quadratus lumborum, which acts as a safety barrier to reduce the risk of visceral damage [23]. Complications associated with QLB are considered minor, and no severe permanent complications have been reported [22, 24, 25].

However, evidence for the analgesic effect of ESPB and QLB is still insufficient, and the results are inconsistent in patients undergoing laparoscopic liver resection. Data on the optimal dose of ropivacaine for ESPB or QLB are limited. Our previous study demonstrated the analgesic effect of ESPB with 40 mL of 0.375% ropivacaine in terms of opioid consumption at 24 and 72 h after laparoscopic liver resection in living liver donors [17]. However, another study conducted at our institution reported only limited opioid-sparing effects in PACU stay despite the use of the same dose of local anesthetics in patients undergoing laparoscopic liver resection [5]. For posterior QLB, we could not verify the analgesic effect of QLB compared with ITM in laparoscopic liver resection for living liver donors [18]. Most recently, we conducted a randomized study comparing the analgesic efficacy of ESPB and QLB using 40 mL of 0.375% ropivacaine after laparoscopic liver resection [10]. The results of this study acknowledged the analgesic potential of QLB but did not demonstrate clear differences in opioid consumption at 24 h after surgery. We considered the reasons for these results to be methodological limitations, including the lack of a comparator and low dose of local anesthetics. To address the limitations of the previous study, we set up a control group of systemic analgesics, and a higher dose of ropivacaine (40 mL of 0.5% ropivacaine) will be employed in this study.

Our study has several limitations. First, the sham procedure is not used for participants in the control group. Second, the optimal dose of ropivacaine for ESPB or QLB has not been determined. Because ropivacaine is mainly eliminated by hepatic metabolism, delayed ropivacaine clearance in patients with liver disease may increase the risk of local anesthetic systemic toxicity (LAST), which may be aggravated after liver resection [26]. Based on our previous study [10], the peak plasma concentrations of ropivacaine were approximately 3-fold below the threshold for systemic toxicity after administration of 40 mL of 0.375% ropivacaine. Therefore, the 40 mL of 0.5% ropivacaine (approximately 15% increased dose) used in this study is not expected to reach the systemic toxicity threshold. Third, to ensure safety, only 20 participants from each block group will be tested for plasma ropivacaine concentration. The sample size of 20 may be too small to discriminate LAST between the groups. Fourth, we plan to measure total plasma ropivacaine concentrations. However, ropivacaine toxicity is dependent on unbound ropivacaine clearance, and the total plasma ropivacaine concentration alone may not be sufficient to determine systemic toxicity [27].

In summary, we anticipate that this trial will provide evidence for the efficacy and safety of ESPB and posterior QLB in patients undergoing laparoscopic liver resection. In addition, our data will provide clinical implications for determining the optimal dose of ropivacaine for ESPB and posterior QLB.

## Trial status

The protocol described in this paper is dated July 22, 2022 (version 1.1). The study protocol is registered with the Clinical Research Information Service on 03 August 2022 (KCT0007599). The first participant was recruited for the trial on August 11, 2022, and recruitment is anticipated to be completed on July 31, 2023.

## Data Availability

The dataset generated by the current protocol will be available from the corresponding author upon reasonable request. The study results will be shared with the public through publication in a peer-reviewed journal.

## Abbreviations

ANOVA: Analysis of variance
BIS: Bispectral index
CRF: Case report form
ESPB: Erector spinae plane block
IV: Intravenous
LAST: Local anesthetic systemic toxicity
MED: Morphine equivalent dose
NSAID: Non-steroidal anti-inflammatory drugs
PCA: Patient controlled analgesia
PROSPECT: Procedure-specific postoperative pain management
QLB: Quadratus lumborum block
SD: Standard deviation
